# Health Tract No. 202

**Published:** 1864-04

**Authors:** 


HEALTH TRACT No. 202.

### THE STOMACH'S APPEAL.

Who but an idiot or some unprincipled servant or recklessly wasteful spendthrift would think of building as large fires in their houses in the April spring-time as in bleak December? And yet ladies and gentlemen, statesmen, philosophers, and scholars of every grade; the judge, the senator, the lawyer, and the clergyman, all commit the more unpardonable folly, unpardonable because it is against light and in favor of the lower instincts and propensities, of not only eating as much as the appetite demands, but of "taking something" to stimulate that appetite to call for more than nature really needs, as the warm weather approaches. The two objects of eating as to men and women are to give vigor to the body and to keep it warm; hence all food contains two principles in greater or less proportions, according to its quality—to wit, nutrition and warmth. We need nourishment all the year round, hence we must all the year round eat food which contains nourishment, that is, the flesh forming principal; but in warm weather the food which contains the most mere fuel, should be to a certain extent curtailed, otherwise we will create too much heat within us, and that is fever, whose victims are counted by millions every year, this excess of heat, this fever being generated by eating food which contains more warmth, more fuel, called carbon by chemists, than the season of the year requires. To a certain extent nature regulates the demand and supply by diminishing the appetite as the warm weather approaches; but many misinterpret her endeavors, and because they find that as the spring comes on their appetites are not as vigorous as they were a few weeks earlier, begin to take alarm, think they are going to get sick, and conclude they certainly will get sick, unless they can get up the appetite of kind winter; hence they begin to take Dutch gin, under the name of Schiedam schnapps, plantation bitters, or cheap whisky, with just enough of colombo root or any other bitter to give it "a trace" of bitter and rob it of the name of "rot-gut" or dirty beer, or ale, or porter, all these things tending to cheat nature into a call for more food than she requires, to impose on the stomach more labor than it can perform, hence laying the ground for summer fevers and dyspepsias, which bring death to thousands every year who might have lived to a good old age had they simply let themselves alone, and like any other dogs or donkeys, or wild beasts, had simply given the stomach rest, and waited for an appetite. The general lessons for the spring are, eat only when you are hungry, and to the extent of satisfying an unstimulated appetite; eat less of carbonaceous food, such as meats, fats, oils, syrups, etc., and more of cooling articles, such as green salads, vegetables, berries, fruits, and whatever has a natural tartness or acidity, there being little or no carbon or heat in them; but they contain as much nutriment as the system requires.

